# Effectiveness of Intergenerational Interaction on Older Adults Depends on Children’s Developmental Stages; Observational Evaluation in Facilities for Geriatric Health Service

**DOI:** 10.3390/ijerph20010836

**Published:** 2023-01-02

**Authors:** Rie Fukuoka, Shinji Kimura, Toru Nabika

**Affiliations:** 1 Department of Community Health and Gerontological Nursing, Faculty of Medicine, Shimane University, Izumo 693-8501, Shimane, Japan; 2 Department of Clinical Nursing, Faculty of Medicine, Shimane University, Izumo 693-8501, Shimane, Japan; 3 Faculty of Medicine, Shimane University, Izumo 693-8501, Shimane, Japan

**Keywords:** intergenerational exchange, older adult, staff, geriatric health services facility

## Abstract

The demand for intergenerational exchange is growing in the world, where the size of a family is becoming smaller. To promote intergenerational exchange, it is important to know whether different stages of child age have different effects. This study aimed to examine effects of intergenerational exchange using children at different developmental stages through a questionnaire survey. The subjects were 296 employees of 116 facilities for geriatric health services that routinely conduct intergenerational exchanges. A questionnaire was designed to ask the employees what effects were observed on old adults and on the employees themselves after intergenerational exchanges were conducted. The results indicated that younger children caused better effects both for older adults and for the employees regarding some of the items, while older children caused equal or lesser effects for all items. It was suggested that the developmental stage of children should be considered according to the purpose of intergenerational exchange.

## 1. Introduction

In developed countries, family size has reduced due to decreasing birthrates as well as due to changes in lifestyles. The shrinkage of family size has led to a reduction of daily communication between children and older adults, which has resulted in adverse effects on both generations, for example, isolation from others in older adults and reduction of ability to establish personal relationships in children [1].

Intergenerational exchange was introduced to compensate for the decreased communication between young and older adults [2,3,4,5,6]. It was shown that interaction with children helped physical functions and ameliorated depression in older adults and expanded personal connections [7,8,9]. It was further suggested that younger children caused a larger impact on older adults through their features attracting adults, i.e., purity and innocence [10,11]. Based on these previous studies, we hypothesized that intergenerational interaction would be more effective with younger children. 

To examine the hypothesis above, the objective of this study was therefore to estimate how the developmental stage of children would influence the effects of intergenerational exchange. For this purpose, we conducted a survey of 116 facilities practicing intergenerational exchange programs.

## 2. Materials and Methods

### 2.1. Definition of Terms

“Intergenerational exchange” means that older adults and children share a time and place to engage in verbal and non-verbal communications.

“The stages of child development” were categorized as Infants, Toddlers, and Young Children (hereinafter referred to as “Infants”) (1 to 6 years old); Lower-Elementary (7 to 9 years old), Upper-Elementary (10 to 12 years old); and Junior-High (13 to 15 years old), using the Japanese developmental classification.

### 2.2. Participants

All facilities that routinely conduct intergenerational exchange programs in the five prefectures of the Chugoku region, centering on Shimane Prefecture, where the authors live, were included. We tried our best to enroll as many participants as possible there. After explaining the study to all facility directors by phone, a questionnaire was sent to them. Questionnaires were sent to 162 facilities for geriatric health services located in the Chugoku area of Japan, of which 116 facilities (71.6%) replied that they had routine intergenerational exchange programs. Facilities for geriatric health services support the independence of older adults in need of nursing care and aim to help them return to their homes. Under medical supervision by a physician, the facility provides nursing and nursing care, rehabilitation by occupational therapists and physical therapists, and daily services such as nutritional management, meals, and bathing. Older adults participating in intergenerational exchanges have the physical and cognitive abilities needed to work with children. Staff members were usually involved with the older adults and were well aware of what they say and do. We conducted a survey of 296 employees who work at those facilities and were involved in intergenerational exchanges. The survey was conducted anonymously so that facilities and individuals were not identified. The survey was conducted on a voluntary basis, and 191 staff members (64.5%) in the facilities responded to our questionnaires. The survey period was from January to June 2016. The protocol of this study was approved by the nursing research ethics committee of Shimane University (#258).

### 2.3. Measurements

The survey form was prepared based on the previous reports [7,10,12,13]. The effects of intergenerational exchange are both physical and non-physical [7]. Physical effects mean whether the physical function improves or not, and we did not measure that in this study. In this study, non-physical effects were measured. Non-physical effects of the intergenerational exchange on the older adults and on the staff were evaluated through responses to questions listed in Table 1. We surveyed the effects that staff members involved in intergenerational exchanges think the elderly feel when they see the expressions, words, and actions of the elderly during intergenerational exchanges. We also surveyed the effects felt by the staff themselves. The effectiveness of intergenerational exchange was evaluated on a six-point scale, from 6 for “very much agree” to 1 for “disagree”. A pre-test was conducted using university employees. We used five examinees, and we ensured that the intent of the questions was communicated to the respondents, the answer choices were appropriate, and the amount and structure of the question were appropriate.

### 2.4. Statistical Analysis

For analysis, the Jonckheere–Terpstra trend test was employed. As no significant difference was found between the male and the female observers, all were analyzed together. *p* < 0.05 was considered statistically significant. Statistical analyses were performed using the SPSS software (v.22, IBM, Armonk, NY, USA).

## 3. Results

### 3.1. Participants

Of the 191 participants who responded to the questionnaires, 25.1% and 74.3% were male and female, respectively. The average (±SD) age was 43.7 (±10.8) years, and the average (±SD) career length was 9.1 (±6.8) years. Job categories were: nurses (30.4%), long-term care welfare workers (38.2%), long-term care staff members (6.3%), physical therapists (4.2%), occupational therapists (3.7%), speech therapists (1.0%), and others (16.2%).

### 3.2. Content of Exchange

The exchange activities included: (1) traditional games in which older adults were actively involved (rice cake pounding, kendama, top spinning, otedama, tea ceremony, etc.), (2) passive games in which older adults were passive (listening to children singing, watching children performing, etc.), (3) games in which older adults and children competed with each other (lottery, rock-paper-scissors, karuta, backgammon, etc.), and (4) games in which older adults and children co-created (singing together, making things together, growing flowers together, cooking together, etc.).

### 3.3. Effects of the Stage of Child Development on the Older Adults (Figure 1)

Significant differences were found on Q1-5 between the Infants and Junior-High categories. Of note, the responses to Q1 and 2 showed a more obvious trend that younger children had better effects. On the other hand, the effects of the Infant and Lower-Elementary categories did not differ significantly in all the questions. Responses to Q6-8 did not differ significantly among the four categories of children. ANOVA with the trend test was also conducted, and the overall trend was the same as that found by the J-T test.

**Figure 1:**
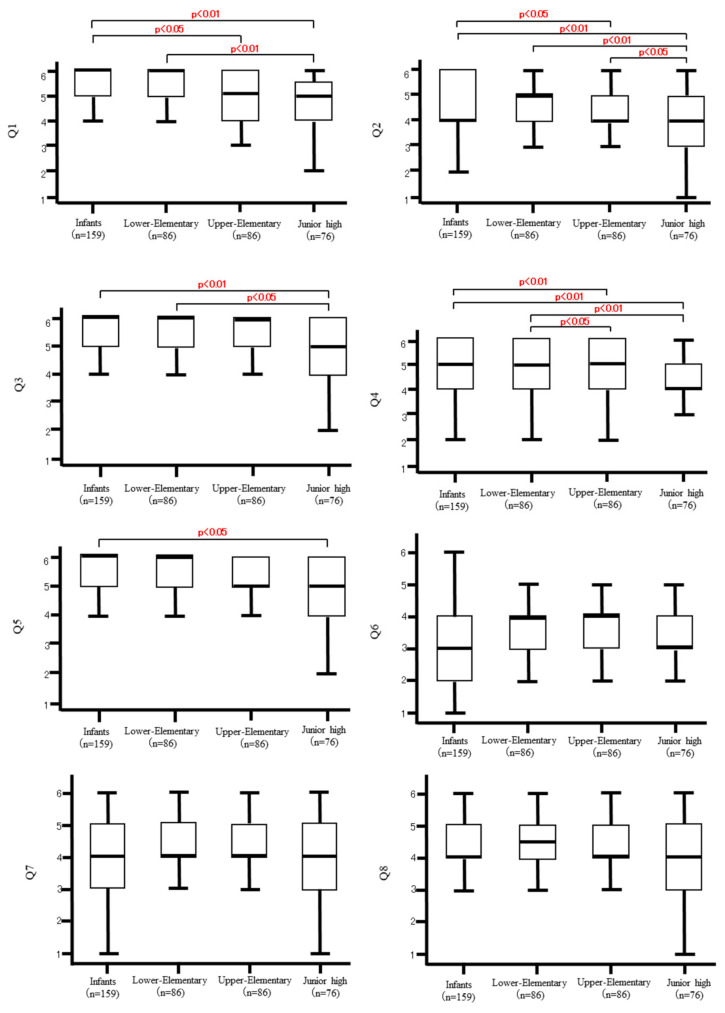
Effects of the stage of child development on the older adult.

### 3.4. Effects of the Stage of Child Development on the Staff (Figure 2)

Significant differences were found on Q1 and 3. The responses to Q1 and 3 showed the same trend as the responses to Q1 and 2 in Figure 1. No significant differences were found between the Infants and Lower-Elementary categories, which was the same as the effects in the older adults (see Figure 1). No significant differences among the four categories of children were observed on Q2 and 4-6. ANOVA with the trend test was also conducted, and the overall trend was the same as that found by the J-T test.

**Figure 2:**
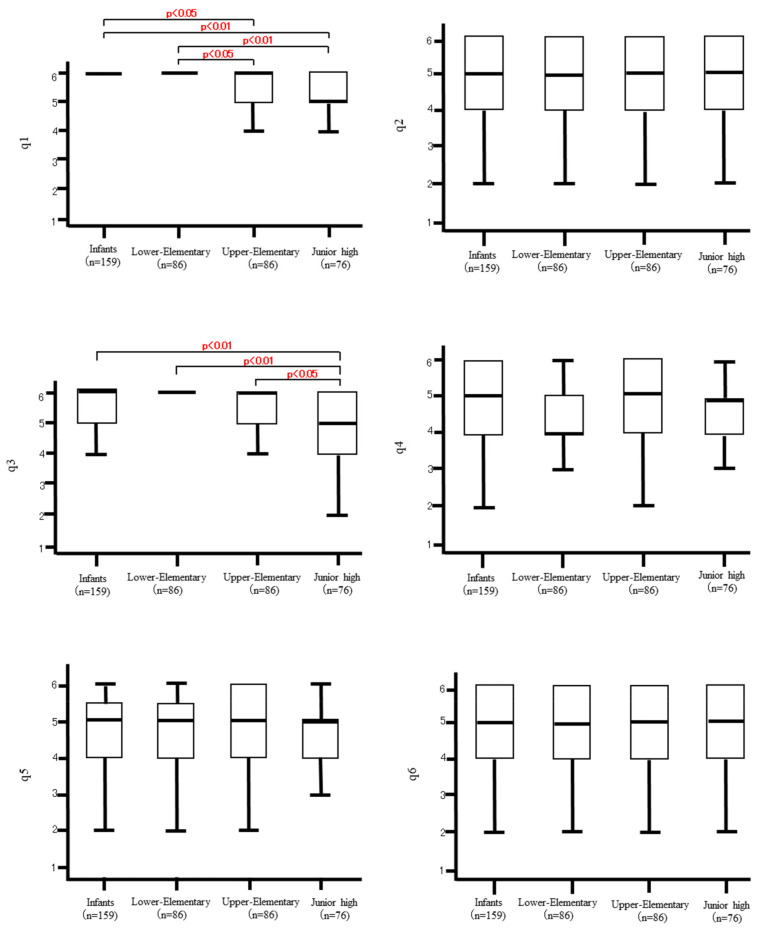
Effects of the stage of child development on the staff.

Box-and-whisker plots: The upper and lower whiskers indicate the maximum and minimum values. The rectangle (box) in the middle represents a quartile. The lower part of the box represents the first quartile (the value of the data in the bottom quarter). The horizontal line in the middle of the box represents the second quartile (the median). The upper part of the box indicates the third quartile (the value of the data in the top quarter).

## 4. Discussion

Younger children were found to be more effective in intergenerational exchange. This could be interpreted as a response to the “baby schema”. The baby schema is a set of infantile physical characteristics such as a large head relative to the body size, larger eyes, chubby cheeks, short and thick limbs, plump body shape, and so on, which are perceived as cute and adorable and trigger positive human emotional responses [14,15]. Cute feelings are positive, non-threatening, moderately arousing, involve motivation to approach the object and watch over it, and seek social interaction, and the effects of cute feelings include attention-getting, smiling, feeling good, wanting to provide care, helping, and healing [14]. The effects of cuteness are said to include attention-getting, smiling, feeling good, wanting to take care of that which elicited the feeling, helping, and healing [16]. We believe that the baby schema/feeling of cuteness elicited positive emotional responses to questions such as “the intergenerational interaction strengthened my feeling of self-esteem”, “softened my feelings”, “made me more motivated”, and “made me more pleasant”.

It was also pointed out that physical characteristics of infants (baby schema) induced caring behaviors [14] and that responses to the baby schema are an important function of human social cognition, which might be the basis of the motivation of those who take care of infants [17]. 

No significant differences were found in all questions regarding the effects of the program by the Infants and Lower-Elementary categories (see Figure 1); older adults as well as staff members of the facilities seemed to feel that children in lower elementary school still kept the baby schema and had not yet lost their infantile thinking and behavior.

In Q6-8, there were no differences in effects based on the age of the children, implying that, for the purpose of obtaining these effects, children of any age were acceptable for the interaction activity.

However, we need to be cautious in employing older children in such interactions. In Q6, “the intergenerational interaction made me more confident in my health”, it was previously pointed out that the older adults rated themselves as healthy when they were able to engage with children [10]. On the other hand, when older adults failed to interact with them in, for example, playing using hands, they felt that their enjoyment of the activity itself was reduced [9]. When older children are employed in the interaction, physical ability will differ largely between the children and the older adults. In the preparation of interaction activities, it is therefore necessary to take into account the cognitive functioning and ADL of the older adults and not to make them lose their confidence in their health.

Older adults also recalled their past life experiences through interaction with children with some nostalgic feelings [10]. In the present study, we showed that, regardless of the age of the children, interacting with children led the older adults to look back upon themselves at a young age.

The age of children caused no difference in the effect to “make the older adult feel more confident in their experience”. An important mental function of older adults is called “generativity,” in which they become more interested in passing the wisdom gained from their past experiences to the next generation [18,19]. Generativity is positively associated with happiness, competence, achievement effort, and trust [20]. The observation in the present study implied that the older adults felt such an interest regardless of the age of the children. 

There were several limitations in this study: First, the effects of the interactions on the older adults were estimated indirectly through observation by staff members of facilities for geriatric health services. Although observation by the staff members might give a more objective view, it would be necessary to verify the effects through direct investigations of the older adults and children. Second, because this study was conducted in the limited region of Chugoku in Japan, the staff and facilities were not representative of those in the whole of Japan. We cannot exclude potential biases due to the region of the survey (western part of the main island of Japan) and randomness of staff members and facilities (only responders were employed; the responders might have greater motivation to use inter-generational exchange programs). It is necessary to expand the scope of the study to include all of Japan in the future. Third, the list of questions was newly developed, and the analyses conducted with single items from the list of questions may have yielded results of low reliability. A more detailed multivariate analysis with more participants and variables is needed.

## 5. Conclusions

Younger children were found to be more effective in intergenerational exchange. The developmental stage of the children should be set according to the purpose of the intergenerational exchange. In addition, when interacting with older children, it is necessary to consider the cognitive functioning and ADL of the older adults so that they do not lose confidence in their health.

## Figures and Tables

**Table 1:** The list of questions.

(**A**) To the older adults:
Q1. The intergenerational exchange promoted my understanding of children.
Q2. The intergenerational exchange strengthened my feeling of self-esteem.
Q3. The intergenerational exchange softened my feeling.
Q4. The intergenerational exchange made me more motivated.
Q5. The intergenerational exchange made me more pleasant.
Q6. The intergenerational exchange made me more confident in my health.
Q7. The intergenerational exchange made me feel more confident in my experience.
Q8. The intergenerational exchange encouraged me to look back on my life.
(**B**) To the staff:
q1. The intergenerational exchange let me find different aspects of older adults.
q2. The intergenerational exchange made me feel more rewarded.
q3. The intergenerational exchange made me smile naturally.
q4. The intergenerational exchange let me expand the range of my work.
q5. The intergenerational exchange gave me an opportunity to reconsider usual nursing care.
q6. The intergenerational exchange increased my respect for older adults.

## Data Availability

Data cannot be shared publicly because of ethical issues. Anyone who wants to access the data should contact the corresponding author.
